# Evaluation of Hematocrit in Adults with Dengue by a Laboratory Information System

**DOI:** 10.1155/2021/8852031

**Published:** 2021-03-27

**Authors:** Duangjai Sahassananda, Vipa Thanachartwet, Putza Chonsawat, Benjamaporn Wongphan, Supat Chamnanchanunt, Manoon Surabotsophon, Varunee Desakorn

**Affiliations:** ^1^Information Technology Unit, Faculty of Tropical Medicine, Mahidol University, Bangkok 10400, Thailand; ^2^Department of Clinical Tropical Medicine, Faculty of Tropical Medicine, Mahidol University, Bangkok 10400, Thailand; ^3^Center Diagnostic Laboratory, Hospital for Tropical Diseases, Faculty of Tropical Medicine, Mahidol University, Bangkok 10400, Thailand; ^4^Pulmonary and Critical Care Division, Department of Medicine, Ramkhamhaeng Hospital, Bangkok 10240, Thailand

## Abstract

The implementation of a laboratory information system (LIS) at the Hospital for Tropical Diseases in Thailand provides valuable medical resources, particularly for dengue. Hematocrit (Hct), which is often derived from hemoglobin (Hgb), is important in the diagnosis and management of dengue. This study aimed to evaluate the Hct value obtained from the LIS automated analyzer. We prospectively enrolled 163 hospitalized adults with dengue, for whom 1,141 real-time complete blood count (CBC) results were obtained via a hematology analyzer and updated in the LIS database. The median (interquartile range (IQR)) duration of analytic turnaround times (TATs) was 40.0 (30.0–53.0) minutes. Linear regression analysis indicated a significant relationship between Hgb and Hct with a coefficient of determination (Pearson's *R*^2^) of 0.92 at red blood cell distribution width (RDW) ≤18, but Pearson's *R*^2^ decreased to 0.78 at RDW >18. The Hct calculated from the three-fold conversion method and from the analyzer had a Pearson's *R*^2^ of 0.92. At Hgb <12 g/dl and ≥16 g/dl, a greater difference between the two Hct values was observed, with median (IQR) differences of −0.8% (−1.9%–0.2%) and 0.8% (−0.1%–1.7%), respectively (*P* value <0.05). In conclusion, the Hgb and Hct of patients with dengue were highly correlated at RDW ≤18. The Hct calculated from the three-fold conversion method and from the analyzer had an excellent relationship, except when the Hgb was <12 g/dl or ≥16 g/dl. Apart from routine CBC evaluation, the LIS could help for accurate data collection in clinical research and development.

## 1. Introduction

The majority of hospitals now actively use a laboratory information system (LIS) linked with the hospital information system (HIS) to gather clinical information [1]. The need for these systems is growing owing to the increased demand for the use of information technology to improve the quality of health services. Laboratory automation helps to improve the processing of laboratory specimens, allowing for convenient patient result verification and decreased turnaround times (TATs), which in turn enables rapid reporting of critical results to improve clinical outcomes [2].

In 2011, a LIS integrated with a HIS was implemented at the Hospital for Tropical Diseases in Bangkok, Thailand. The LIS in our hospital, particularly for the automation of the hematology analyzer, was implemented to obtain real-time complete blood count (CBC) results, system security, data entry control, medical reports, and data retrieval and storage to improve clinical care and ultimately reduced the probability of human errors [3–6].

Dengue is a major public health problem in tropical countries, particularly in Southeast Asian countries, and has a negative economic and health impact on low- and middle-income countries [7]. An increasing incidence of dengue and its severity was reported in adults [8]. Approximately two-thirds of adults with dengue presented with atypical manifestations such as upper respiratory tract infection and acute gastroenteritis, and approximately 10% of hospitalized patients developed fatal dengue complications, including severe plasma leakage, resulting in shock or respiratory distress, organ involvement, and bleeding [8, 9].

Aside from the hematological features, including hemoconcentration, leukopenia, and thrombocytopenia, hematocrit (Hct) is also important for diagnosis and case management in dengue [9]. Hct is the ratio of packed red blood cell volume to total blood volume estimated via microhematocrit, CBC, or hemoglobin (Hgb) tests [10]. However, each method has limitations that affect the accuracy of the Hct value, such as the blood sample preparation technique and variability in red blood cell (RBC) size and shape. In dengue, polychromasia, leukopenia, plasma leakage, and the blood collection technique and analysis could lead to falsely high or low Hct [11].

Hct and Hgb levels are analyzed as part of a CBC. Hgb is measured directly from the analyzer, while Hct is calculated by multiplying the mean cell volume (MCV) by the RBC count. A significant correlation between Hct and Hgb was previously reported with a Pearson's *R*^2^ of 0.99 [12]. Hct also showed a good relationship with Hgb in patients with malaria, except when Hgb was <11 g/dl [13, 14]. In our practice, Hct is calculated from Hgb using three-fold conversion (that is, Hgb = Hct/3) due to its convenience [15]. A previous study showed that the three-fold conversion was valid, but the results were slightly lower than those from an automated hematology analyzer [14].

Anemia occurring with conditions causing low MCV, such as thalassemia and iron deficiency, might lead to falsely low Hct results [16, 17]. We hypothesized that Hct might be positively associated with Hgb unless the patient suffered from anemia. Therefore, we conducted a prospective study aimed to evaluate the Hct value obtained from the LIS automated analyzer among adults with dengue.

## 2. Materials and Methods

### 2.1. Ethical Considerations

This study was conducted according to the Declaration of Helsinki. The protocol of this study was approved by the Ethics Committee (MUTM 2013-008-04) of the Faculty of Tropical Medicine, Mahidol University, Bangkok, Thailand. Written informed consent was obtained from all patients or their respective guardians for patients aged less than 18 years.

### 2.2. Materials

This study was conducted following the Standards for the Reporting of Observation Studies in Epidemiology (STROBE) statement [18]. This was a prospective study that included hospitalized patients with confirmed dengue between October 2015 and January 2017. The inclusion criteria were (1) aged at least 15 years; (2) presented with acute febrile illness defined by fever less than 7 days without organ-specific symptoms; and (3) viral dengue infection confirmed via positive results from one of the following: (a) a viral nucleic acid assay using reverse-transcription polymerase chain reaction (RT-PCR), (b) a microneutralization test, or (c) dengue-specific IgM and IgG antibodies using an enzyme-linked immunosorbent assay (ELISA). The exclusion criteria were (1) a history of underlying medical illness, for example, diabetes mellitus, hematologic diseases, chronic kidney disease, or malignancy; (2) the presence of a comorbid infection, such as malaria, rickettsiosis, leptospirosis, or bacterial infection; and (3) receiving blood transfusion. Data were anonymized before analyses.

### 2.3. Methodology

**Figure 1:**
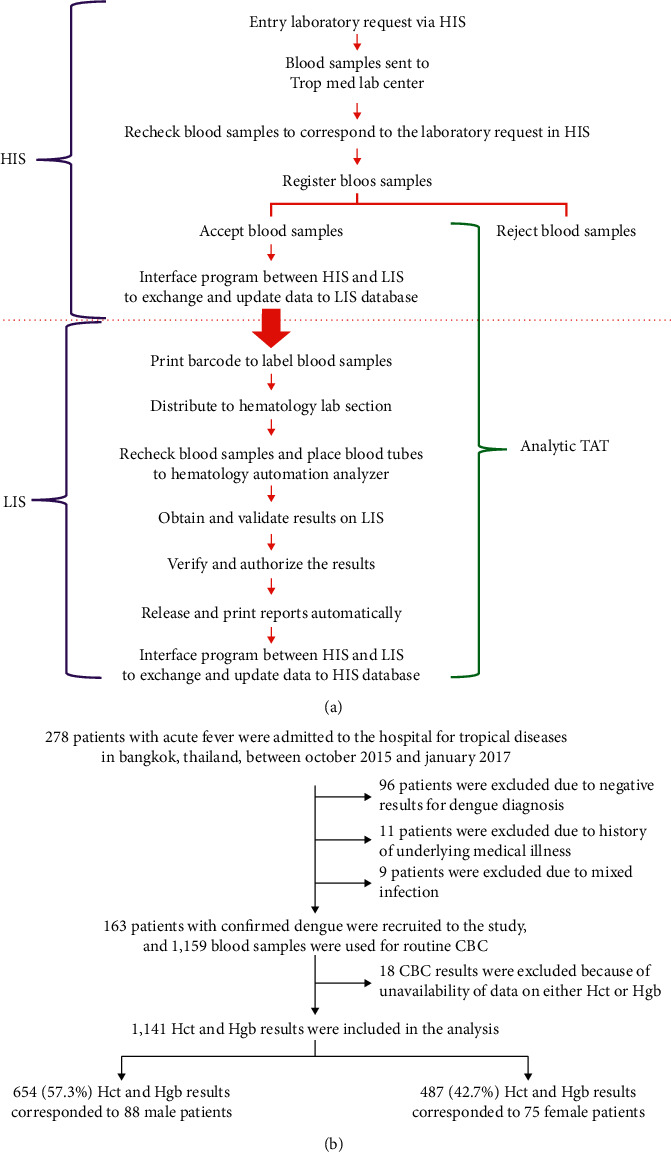
Flow diagram of (a) the study system and (b) patient recruitment.

LIS plays a role in the use and archiving of laboratory test results by increasing their accuracy and decreasing human errors [2, 19]. The LIS in our hospital has a new design workflow using HCLAB (Sysmex Corporation, Kobe, Japan), which is a graphical user interface that allows cross-referencing with the HIS. The LIS is operated using client-server technology. Whole blood samples for routine CBC were collected in dipotassium ethylene-diamine-tetra-acetic acid anticoagulant tubes (Improvacuter, Hamburg, Germany) and immediately submitted to the central laboratory of the hospital via the pneumatic tube system. Before further processing, all samples were anonymized and labeled with unique barcode identifiers. The study system, including workflow and analytic TAT, is shown in Figure 1(a). The analytic TAT was defined as the time from accepting blood samples in the laboratory to the time of reporting results simultaneously in the LIS and HIS.

### 2.4. Hematology Laboratory Complete Blood Count Measurements

All blood samples were tested in our hematology laboratory using a Siemens ADVIA 120 (Siemens Healthcare Diagnostics, Eschborn, Germany) as the routine automated analyzer. The analyzer was run following a standard operating procedure and checked daily using quality control samples. The automated analyzer processed the samples for all hematology parameters, including RBC, Hgb, Hct, MCV, mean corpuscular hemoglobin (MCH), mean corpuscular hemoglobin concentration (MCHC), red blood cell distribution width (RDW), white blood cell count (WBC), platelet count (PLT), and mean platelet volume.

The analyzer assessed RBCs using flow cytometry and light scattering (laser). Each cell was analyzed for RBC volume and Hgb concentration based on the degree of light scattering. Hgb was determined via colorimetric measurement after chemical lysis of RBCs. The analyzer calculated the Hct as the number of RBCs per MCV, i.e., Hct = (RBC × MCV)/10 [20]. All Hct and Hgb values obtained simultaneously from blood samples from the dengue patients during hospitalization were reported to the LIS. Patient data were searched, queried, and retrieved from the database for analysis.

### 2.5. Statistical Analysis

Numerical data were expressed as the mean and standard deviation (SD) and were compared using student's *t*-test for comparisons between 2 groups. In cases of skewed distribution, data were expressed as medians and interquartile ranges (IQRs), the Kruskal–Wallis test was used for comparisons of more than 2 groups, and post hoc analysis was performed via the Mann–Whitney *U* test for pairwise comparisons. Bonferroni's correction was used to adjust significance levels obtained from the series of Mann–Whitney test results by multiplying unadjusted significance values by the number of comparisons. Categorical data were expressed as frequencies and percentages and were analyzed using the chi-squared test or Fisher's exact test as appropriate. The paired determination of Hgb and Hct was presented as a scatter plot and was analyzed via linear regression analysis with the least squares method. Pearson's *R*^2^ was used as a measure of correlation between Hgb and Hct levels. The Bland–Altman method was used to assess the agreement between Hct calculated from three-fold Hgb and Hct measured by the automated hematology analyzer. The difference between the two Hct measures was calculated for 95% limits of agreement by the mean difference ±1.96 SD. Statistical significance was obtained using two-sided tests with a *P* value of <0.05. All statistical analyses were performed using SPSS for Windows 18.0 (IBM Corp., Chicago, IL).

## 3. Results

Of the 278 patients with dengue, 115 patients were excluded. Finally, 163 patients with confirmed viral dengue infection were included (Figure 1(b)); from these patients, 1,159 blood samples were used for CBC. A total of 18 CBC results were excluded due to the unavailability of either Hct or Hgb results. Thus, 1,141 paired Hct and Hgb results, including 654 (57.3%) data points from male patients and 487 (42.7%) data points from female patients, were available for analysis.

### 3.1. Baseline Characteristics and Red Blood Cell Parameters among Adults with Dengue

The median (IQR) age of the 163 patients with dengue was 24.0 (19.0–36.0) years. There were 88 male patients (54.0%) and 75 female patients (46.0%). The severity of dengue was classified using the World Health Organization (WHO) 2009 criteria [9] as dengue without warning signs in 8 patients (4.9%), dengue with warning signs in 125 patients (76.7%), and severe dengue in 30 patients (18.4%). Severe organ involvement due to dengue occurred in 19 patients (63.3%), dengue shock syndrome in 17 patients (56.7%), and severe clinical bleeding in 8 patients (26.7%). There was no significant difference in dengue severity between male and female patients (Table 1).

The median (IQR) duration of hospitalization was 15.0 (13.0–17.0) days. The median (IQR) duration of the analytic TAT was 40.0 (30.0–53.0) minutes and was similar among male and female patients. Additionally, the female patients had significantly lower Hgb, Hct, RBC, MCV, MCH, and MCHC levels compared to the males. In contrast, the female patients had a significantly higher PLT level compared to the males (Table 1).

### 3.2. Relationship between Hematocrit and Hemoglobin among Adults with Dengue

**Figure 2:**
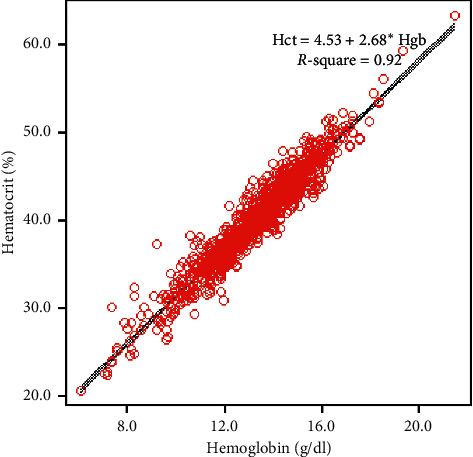
Scatter plots of the 1,141 paired measurements of hematocrit and hemoglobin with best-fitting line.

**Figure 3:**
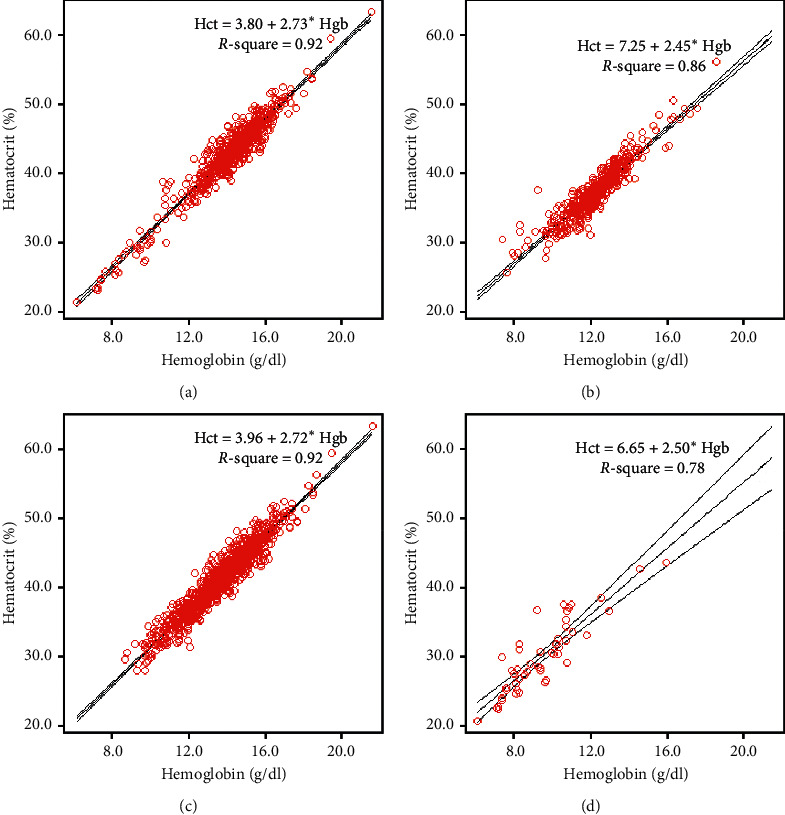
Paired hematocrit and hemoglobin of dengue patients by sex and red cell distribution width (RDW). Scatter plots of the (a) 654 paired measurements of hematocrit and hemoglobin from 88 men with the line of best fit; (b) 487 paired measurements of hematocrit and hemoglobin from 75 women with the line of best fit; (c) 1,092 paired measurements of hematocrit and hemoglobin from patients who had RDW ≤18 with the line of best fit; (d) 49 paired measurements of hematocrit and hemoglobin from patients who had RDW >18 with the line of best fit.

Analysis showed significant correlation between Hgb and Hct with a coefficient of determination (Pearson's *R*^2^) of 0.92. The line of best fit via linear regression analysis had a regression coefficient of 2.68. Therefore, the following equation from the regression line was applied: Hct (%) = 4.53 + 2.68 × Hgb (g/dl) (Figure 2). When the paired Hgb and Hct were divided according to those obtained from male and female patients, a significant relationship between Hgb and Hct was still observed with a coefficient of determination (Pearson's *R*^2^) of 0.92 for males (Figure 3(a)) and 0.86 for females (Figure 3(b)). As Hct was calculated from the MCV and RBC count, RDW might have an effect on the Hct level. Therefore, paired Hgb and Hct results were divided according to RDW level. With a cutoff value of 18 for RDW, 49 of the 1,141 (4.3%) paired Hgb and Hct data had an RDW >18. A significant relationship between Hgb and Hct was observed with a coefficient of determination (Pearson's *R*^2^) of 0.92 when the RDW was ≤18 (Figure 3(c)), but the correlation decreased with a Pearson's *R*^2^ of 0.78 when the RDW was >18 (Figure 3(d)).

### 3.3. Relationship between the Hematocrit Value Obtained from the Automated Analyzer and Estimated from a Three-Fold Conversion of the Hemoglobin Value among Adults with Dengue

**Figure 4:**
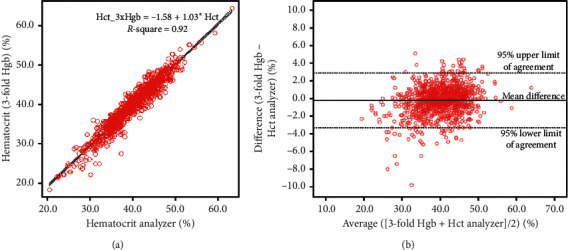
Paired hematocrit of dengue patients calculated from the three-fold conversion and the analyzer. (a) Scatter plots of the 1,141 paired data with hematocrit calculated from the three-fold conversion and the analyzer with best-fitting line. (b) Bland–Altman plot for agreement of data from hematocrit calculated from the three-fold conversion and the analyzer. The mean of the two measurements (horizontal axis) was plotted against the difference between the hematocrit calculated from the three-fold conversion and the analyzer (vertical axis).

**Figure 5:**
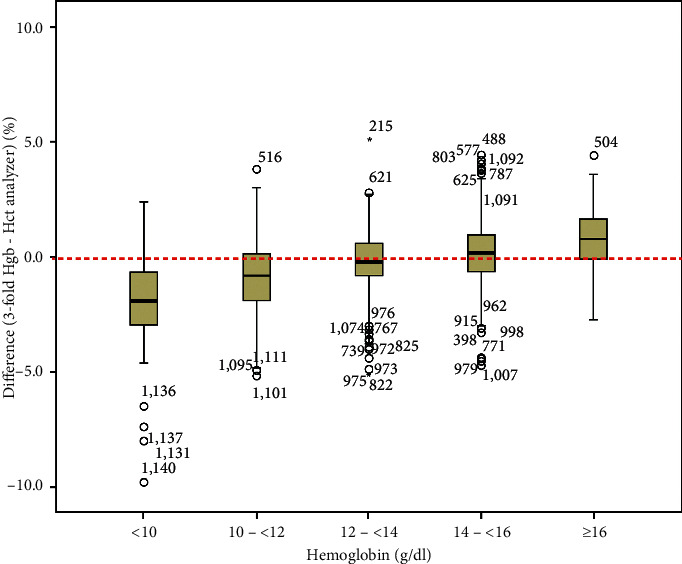
Difference between hematocrit calculated from the three-fold conversion and the analyzer by hemoglobin levels. Data are presented in box and whisker plots with median (horizontal line), interquartile range (box), and maximum values within 1.5 of interquartile range (whiskers).

We investigated whether the Hct value calculated via the three-fold conversion was different from that obtained from the analyzer. A significant relationship between the Hct calculated from the three-fold conversion and from the analyzer was observed with a coefficient of determination (Pearson's *R*^2^) of 0.92. The line of best fit showed a regression coefficient for linear regression of 1.03. Therefore, the following equation from the regression line was applied: Hct calculated from three-fold conversion of Hgb (%) = −1.58 + 1.03 × Hct reported from the analyzer (%) (Figure 4(a)). The Bland–Altman plot showed that the mean difference between Hct calculated via the three-fold conversion and the analyzer was −0.2%, and the 95% limits of agreement were −3.31%–2.90% (Figure 4(b)). The difference between Hct calculated via the three-fold conversion and via the analyzer was observed in relation to different levels of Hgb. The Hgb measurement was divided into levels of <10 g/dl, 10–<12 g/dl, 12–<14 g/dl, 14–<16 g/dl, and ≥16 g/dl. The median (IQR) differences between Hct calculated via the three-fold conversion and the analyzer were −1.9 (−3.0–−0.6)%, −0.8 (−1.9–0.2)%, −0.2 (−0.8–0.6)%, 0.2 (−0.6–1.0)%, and 0.8 (−0.1–1.7)%, respectively (Figure 5). Using Bonferroni's correction, a significant difference among all possible pairwise comparisons of the difference between Hct calculated via the three-fold conversion and Hct measured with the analyzer was observed with a *P* value <0.001, except for the pairwise comparison between the groups with Hgb level 14–<16 g/dl and ≥16 g/dl, which showed a significant difference at a *P* value of 0.003.

## 4. Discussion

Information management by implementing a LIS integrated with a HIS has the benefits of increased healthcare service delivery, enhanced monitoring, reduced medical errors, decreased rate of data redundancies, provided timely results, and supported academic research [1]. In dengue, accurate and reproducible Hct measurement would be important for making decisions about fluid management [9]. However, the traditional centrifugation method used to prepare samples to measure Hct level showed overestimated results due to increased plasma volume trapped in the red cell column, particularly in patients with anemia [15, 21]. In point-of-care measurements for Hct level, the relative difference was 4%, with a difference above the Clinical Laboratory Improvement Amendments (CLIA) criteria of 28% in paired samples when compared to central laboratory measurements [22].

A previous study showed that the Hgb level was relatively stable after venipuncture, but the Hct level increased with the blood sample age [15]. Using point-of-care measurements for Hgb level, the relative difference was 8%, with a difference above the CLIA criteria of 65% in paired samples when compared to central laboratory measurements [22]. Thus, central laboratory measurement of Hct would be important for dengue diagnosis and clinical management [8, 9]. LISs play an important role in terms of requesting and collecting samples and producing accurate, precise, and timely results that ultimately lead to better healthcare service [23, 24].

A significant relationship between Hgb and Hct reported from the analyzer was observed with a coefficient of determination (Pearson's *R*^2^) of 0.92 in our patients with dengue, and this was similar to that reported in a previous study in trauma patients, which yielded a Pearson's *R*^2^ of 0.99 between Hct and Hgb [12]. This might be due to the short duration of analytic TAT (40 minutes). Over the past 6 decades, automated hematology analyzers showed to enhance the speed, accuracy, precision, and interpretation of test results [24]. Information technology positively impacts healthcare by helping to improve the quality and efficiency of numerical data management and delivery of clinical care [1]. In women with dengue, the relationship between Hct and Hgb showed a Pearson's *R*^2^ of 0.86, which was lower than that for men at 0.92. This difference might be due to the different impact of sex hormones on erythropoiesis between sexes [25].

As Hct was calculated from the MCV and RBC count, high RDW might have an effect on the Hct level. In our study, the Pearson's *R*^2^ decreased to 0.78 for the relationship between Hct and Hgb, which was acceptable when the RDW was >18. A previous multicenter study on normal Hct in North America reported that the Hct level derived from the three-fold conversion method could be used to eliminate overestimation of the Hct level from the traditional centrifugation method [26]. In our study, the Hct calculated from the three-fold conversion and from the analyzer showed a significant relationship, with a Pearson's *R*^2^ of 0.92. Moreover, there was a significant concordant correlation coefficient between the Hct level calculated from three-fold Hgb and Hct reported from the analyzer. The Bland–Altman plot showed good agreement between the Hct level calculated from the three-fold conversion and from the analyzer, but the Hct level calculated via the three-fold conversion method tended to be slightly lower by 0.2%. This finding was similar to that in a previous study in malaria patients [14]. The differences between Hct calculated from the three-fold conversion method and from the analyzer had clinical significance when the Hgb was <12 g/dl or ≥16 g/dl.

Our study has limitations. The single-center setting might have led to some bias. The results might not be generalized to the population because only patients with dengue were included. However, Hct in patients with dengue might be normal, high, or low, making them suitable for the study.

## 5. Conclusions

In conclusion, this study utilized data from the LIS database to evaluate the Hct and Hgb levels among patients with dengue. The Hct and Hgb levels of patients with dengue were significantly correlated, with a high coefficient of determination, except when the RDW was >18. The Hct calculated via the three-fold conversion method had an excellent relationship with the Hct calculated via the automated hematology analyzer, except when the Hgb was <12 g/dl or ≥16 g/dl. Apart from routine hospital applications to improve accurate data collection, the LIS database could be helpful for clinical research.

## Figures and Tables

**Table 1:** Baseline characteristics and red blood cell parameters among 163 patients with confirmed viral dengue infection.

Characteristics	All	Men (*n* = 88)	Women (*n* = 75)	*P* value
	Median (IQR)	Median (IQR)	Median (IQR)	
Age (years)	24.0 (19.0–36.0)	23.0 (20.0–33.5)	26.0 (19.0–39.0)	0.254
Dengue severity				
Nonsevere dengue, *n* (%)	133 (81.6)	71 (80.7)	62 (82.7)	0.902
Severe dengue, *n* (%)	30 (18.4)	17 (19.3)	13 (17.3)	
Analytic TAT (min)	40.0 (30.0–53.0)	40.0 (30.0–53.0)	41.0 (30.0–55.0)	0.440
Red blood cell parameters				
Hgb, mean (SD) (g/dl)	13.4 (1.9)	14.2 (1.8)	12.3 (1.4)	<0.001
Hct, mean (SD) (%)	40.3 (5.2)	42.6 (5.1)	37.3 (3.8)	<0.001
RBC (×10^12^/l)	4.9 (4.5–5.4)	5.2 (4.8–5.8)	4.6 (4.3–5.0)	<0.001
MCV (fl)	82.5 (76.3–88.0)	83.0 (77.0–88.8)	81.7 (76.1–87.3)	0.002
MCH (pg/RBC)	27.6 (25.3–29.4)	28.0 (25.7–29.9)	27.0 (25.1–29.1)	<0.001
MCHC (g/dl of RBC)	4.9 (4.5–5.4)	5.2 (4.8–5.8)	4.6 (4.3–5.0)	<0.001
RDW (%)	14.2 (13.7–15.2)	14.3 (13.8–15.2)	14.2 (13.6–15.1)	0.120
WBC (×10^3^/*μ*l)	4.8 (3.0–6.4)	4.8 (3.1–6.6)	4.7 (2.9–6.2)	0.121
PLT (×10^3^/*μ*l)	95.0 (53.0–178.0)	88.0 (50.0–169.0)	103.0 (58.0–200.0)	0.027
MPV (fl)	8.0 (7.3–9.3)	8.1 (7.3–9.6)	7.9 (7.3–9.0)	0.091

Hgb = hemoglobin; Hct = hematocrit; RBC = red blood cell count; MCV = mean cell volume; MCH = mean corpuscular hemoglobin; MCHC = mean corpuscular hemoglobin concentration; RDW = red cell distribution width; WBC = white blood cell count; PLT = platelet count; MPV = mean platelet volume; TAT = turnaround time.

## Data Availability

The data used to support this study are available from the corresponding author upon request. The data are not publicly available because they contain information that could compromise the privacy of research participants.
